# Crystallographic hallucinations

**DOI:** 10.1107/S2053229622000511

**Published:** 2022-01-27

**Authors:** Brian H. Toby

**Affiliations:** a Argonne National Laboratory, 9700 South Cass Avenue, Darien, IL 60439, USA

**Keywords:** psilocybin, Rietveld, pharmaceutical, psychedelic, qu­anti­tative phase analysis

## Abstract

In a detailed powder diffraction study of the structural forms of psilocybin, Sherwood *et al.* [*Acta Cryst.* C**78**, 36–55] cast doubt that any other anhydrous polymorphs could exist.

Psilocybin was of great inter­est to some of my contemporaries during my student days, but not due to its crystal structure, at least not directly. At present, due to the study of hallucinogens as possible treatment agents for various mental illnesses, their use has become a topic of significantly renewed inter­est (Gill *et al.*, 2020 ▸; Sherwood & Prisinzano, 2018 ▸). As is well known in the pharmaceutical industry, crystallographic characterization of materials is crucial in order to understand the bioavailability of a drug (Byrn *et al.*, 1999 ▸). Structural knowledge is thus highly important for the potential use of psilocybin.

In their detailed study of the structural forms of psilocybin reported recently in *Acta Crystallographica Section C*, Sherwood *et al.* (2022 ▸) solve two novel anhydrous psilocybin structures *ab initio* from high-resolution synchrotron and from laboratory X-ray powder data, respectively. While attempts to directly crystallize the anhydrous forms were attempted, they could only produce the phases by removing water from a hydrated form. The hydrated phase forms single crystals and has a determined structure, but dehydration destroys these crystals, producing crystalline powders. To confirm the quality of the powder structures and review the hydrogen-bonding motifs, they performed computations with plane-wave density functional theory (DFT). A second form of DFT was used in ‘gas phase’ computations to compare the energetics of the psilocybin conformations for isolated mol­ecules for their two new polymorphs and the previously-known hydrated form.

While the very high quality of the fits between the observed and calculated patterns would already give one confidence in their results, their verification that that calculations from theory demonstrate that the structures represent energetic minima boosts confidence in their results manyfold. However, it had been reported in the patent literature that different polymorphs exist. To address this, Sherwood *et al.* (2022 ▸) performed Rietveld fits on data from a total of 24 different samples, either with contemporary data collection or with digitized powder diffraction patterns taken from the literature. They show that *all* these patterns are consistent with their two new anhydrous structures and the known hydrous form, sometimes as mixtures and sometimes where intensities require correction for significant preferred orientation. This texture is not at all surprising considering the highly anisotropic crystal habits they see in the powders. Furthermore, they made significant efforts to coax psilocybin to crystallize in other structural forms, but to no avail. Based on all this, the previous assignment of other phases for anhydrous psilocybin seems highly unlikely and this careful work casts doubt that any other anhydrous polymorphs could exist.

This *tour de force* study is one that deserves exposure in the classroom, so that crystallography students may see how powder diffraction crystallography can rise to the gold standard level that we expect from single-crystal analysis. In fact, it is worth noting that the agreement seen between DFT and the powder–synchrotron structure is far better than the DFT–single crystal agreement. The impact of this work on patents issued with incorrect crystallography is something to be resolved in courts, not the scientific literature, but I have no doubt that a number of scientific managers are currently considering the potential impact that less-than-careful crystallography might have on their organization’s intellectual property collections.

The compelling structural validation work by Sherwood *et al.* (2022 ▸) demonstrates the great value of DFT as a partner tool to crystallography. As I write this, on the eve of International Holocaust Remembrance Day, thinking about DFT raises for me thoughts on another topic of contemporary worldwide interest – if not contention – which is on the value in opening borders for immigration. It is worth remembering that the life of Walter Kohn, who won the Nobel Prize for his development of DFT, was saved by the British *Kindertransport*, which resettled children in the UK even when their parents could not cross borders. As a child, Walter and his sister Minna were able to leave Austria for Britain and thus survived World War II. Walter was educated in Canada and eventually immigrated to the United States. Walter’s work gave the world this highly useful approach to quantum theory, but he never saw his parents again; they died in Auschwitz (Kohn, 1998 ▸; https://www.notablebiographies.com/supp/Supplement-Ka-M/Kohn-Walter.html).

As a final point, with the ubiquitous availability of fast computers and with easier to learn codes (some open source), DFT is an increasingly accessible tool for crystallographers. Even with a good-quality single-crystal structure, verification of hydrogen-bonding networks is a worthwhile exercise. With powder diffraction crystallography, structural validation becomes even more valuable.
